# Effects of Gastric Irrigation on Bacterial Counts before Endoscopic Submucosal Dissection: A Randomized Case Control Prospective Study

**DOI:** 10.1371/journal.pone.0065377

**Published:** 2013-06-07

**Authors:** Hirohito Mori, Hideki Kobara, Kazi Rafiq, Noriko Nishiyama, Shintaro Fujihara, Makoto Oryu, Tsutomu Masaki

**Affiliations:** 1 Department of Gastroenterology and Neurology,, Faculty of Medicine, Kagawa University, Kagawa, Japan; 2 Department of Pharmacology, Faculty of Medicine, Kagawa University, Kagawa, Japan; National Cancer Center, Japan

## Abstract

**Objective:**

The antiseptic effect of gastric irrigation before endoscopic submucosal dissection (ESD) has not yet been reported. The aim of the randomized prospective study is to evaluate the antiseptic effects of gastric irrigation of saline solution before ESD by evaluating bacterial count.

**Methods:**

This prospective randomized controlled trial included 50 patients diagnosed with early gastric cancer who were randomly divided into 2 groups (25 patients in each group) by using the opaque envelope method: the clean group (irrigation with 2 L saline solution before ESD) and the regular group (no irrigation). The gastric juice was collected and cultured before ESD. The entire stomach was irrigated using a water jet attached to an endoscope. After ESD with resection and removal of the tumor specimen, a postoperative culture of the gastric juice was obtained using the same method as the preoperative culture.

**Results:**

The mean log bacterial count of the post-gastric irrigation gastric juice was 5.08±0.75 in the regular group and 1.86±0.86 in the clean group. The difference in the bacterial counts was significant between the groups (*P* = 0.0004). The difference in the white blood cells (WBC) count on POD 1 was significant (*P* = 0.044). WBC count on POD 2 did not significantly differ between the groups (*P* = 0.3). The difference in the body temperature (BT) on POD 1 was significant (*P* = 0.017), On POD 2 the BT was not significant between the groups (*P* = 0.5). On POD 1, 88% of the patients in the regular group and 16% of the patients in the clean group had mild to moderate spontaneous pain (*P* = 0.0026). On POD 2 the proportion with mild to moderate spontaneous pain was 36% and 24% in the regular group and the clean group, respectively (*P* = 0.1).

**Conclusion:**

Pre-ESD gastric irrigation with saline solution is effective and feasible for suppressing infection during the ESD procedure with favorable clinical outcomes.

**Trial registration information:**

The university hospital medical information network (UMIN) #000008691.

## Introduction

In surgery, hand washing is known to minimize contamination in the operative field and reduce the indigenous bacterial volume exponentially, even in cases when surgical gloves break. The search for the most advanced means of surgical disinfection is ongoing. The methods reported in recent years include scrubbing with sterile water using a sterile culture brush to disinfect the skin and waterless rubbing using regular soap and fast-drying disinfectant; these methods are recommended by the United States Center for Disease Control (CDC) [1]. During surgical maneuvers, such as skin incision and the approach to target organs, the indigenous bacteria, *Mycobacterium tuberculosis*, filamentous fungi, spore-forming bacteria, and viruses are all potentially pathogenic and can cause infections. During endoscopic submucosal dissection (ESD), an endoscope is inserted into the stomach through the mouth, which results in the inevitable exposure of the dissection site to oral bacteria, which may led to infection. However, no reports exist concerning gastric irrigation before ESD, as intraluminal treatment in the stomach is a semi-closed system where gastric acid is present. Therefore, the validity of irrigation with a saline solution in ESD has yet to be confirmed.

A frequent complication during and after ESD is perforation [2], which can often be treated with conservative therapy by closing the perforation with a clip [3]. If it is proven that gastric irrigation can reduce bacterial counts, it can then be expected that intra-abdominal infection might be more easily suppressed by gastric irrigation before procedure without using antibiotics, even when complications such as perforation occur. The present study was a prospective randomized controlled trial of the effects of gastric irrigation with 2 L of saline solution before ESD on gastric bacterial counts.

## Patients and Methods

The protocol for this trial and supporting CONSORT checklist are available as supporting information; see Checklist S1 and Protocol S1.

This prospective randomized study included 50 patients who were diagnosed with early gastric cancer at Kagawa University Hospital from June to November 2012.

We conducted a pilot study with 8 patients who underwent ESD for early gastric cancer after receiving approval from the institutional ethics committee. Among the 8 patients, 4 patients were irrigated with 2 L of saline solution before ESD techniques and 4 were not. We calculated the sample size from the pilot study.

**Figure 1:**
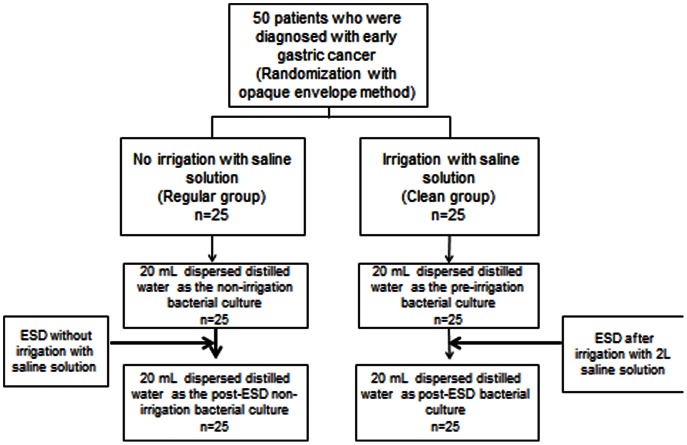
Allocation flow chart.

An opaque envelope method was used to randomly divide the subjects into a clean group; in which irrigation was performed before ESD, and a regular group; in which irrigation was not performed. Each group contained 25 patients. All of the patients began a 30 mg daily dose of a proton-pump inhibitor (esomeprazole) on the day prior to ESD. The allocation flow chart for this study is shown in Figure 1. The randomization was achieved using sealed and numbered envelopes, as prepared previously by Dr. M. O. The randomization code was not broken until the study was completed. All of the investigators attended a study meeting before the study and received instructions about the methods for gastric irrigation, measuring the distilled water dispersion and the collection of gastric juice with a sterile culture tube. ESD was performed by 1 of 5 endoscopists. None of the endoscopists were informed about this study, but each endoscopist was informed on how to perform the irrigation and was blinded to the randomization process to avoid any bias. All bacterial cultures were performed by a bacteriologist (Dr. N. N.) who was blinded to the randomization process and did not know which patients received irrigation. At the end of the study, the data were analyzed by Dr. H. M. and Dr. K. R. in a blinded manner to avoid bias and prepared the manuscript.

At the beginning of the ESD procedure, 20 mL of distilled water was dispersed onto the gastric wall, and 20 mL of gastric juice was collected in a sterile culture tube for evaluation as the pre-procedure bacterial culture (37°C, 48 hours). After that, in clean group, irrigation was performed throughout the stomach with 2 L of saline solution using a water jet attached to an endoscope (GIF Type Q260J, Olympus, Tokyo, Japan). After completion of the ESD and removal of the resected tumor, 20 mL of distilled water was again dispersed onto the gastric wall, and 20 mL of gastric juice was collected in a sterile culture tube as the post-ESD culture. The sterile culture tube was passed through the working channel. We counted the number of times the endoscope was withdrawn and recorded the normal saline volume that was used to wash the stomach during ESD (except for irrigation). White blood cell (WBC) counts and C-reactive protein (CRP) levels were measured on the day before ESD and on the days 1 and 2 post-ESD. The body temperature (BT) was checked on the day before ESD and on the days 1 and 2 post-ESD. The physical examination included an assessment of spontaneous abdominal pain using a 4-level visual analog scale (VAS): VAS-0 represented no spontaneous pain, 1 represented mild spontaneous pain, 2 represented moderate spontaneous pain, and 3 represented severe spontaneous pain. In addition, a chest X-ray was taken the day after ESD to check for aspiration pneumonia. The procedures described above were the same in both groups. The endoscopes were treated with high-level 2.4% glutaraldehyde disinfectant (Cidex, Johnson & Johnson, Irvine, CA) and dried after every procedure.

### Ethical Statement

This prospective clinical study was conducted with pre-approval by the institutional ethics committee of Kagawa university hospital, Kagawa, Japan and was enrolled with the university hospital medical information network (UMIN) #000008691. And the study began after we obtained informed consent to patients by written form.

### Operative Devices

We used the Olympus GIF Type Q260J, Olympus GIF Type H260Z and Olympus GIF Type XP260NS endoscopes. All of the endoscopes were sterilized with EtO gas. The operations were aseptically performed with a flexible endoscope. We used a Dual knife (KD-650L, Olympus) and an IT knife 2 (KD-611L, Olympus) for the incisions. We used an ERBE VIO300D incisional generator and an Olympus UCR as the CO_2_ insufflation device.

### Outcomes

The primary outcome was the difference in pre-ESD and post-ESD gastric juice culture bacterial counts between the clean and the regular group.

The secondary outcomes are as the following: (1) WBC, CRP and BT values on days 1 and 2 post-operation. (2) Spontaneous pain level VAS scores just after the operation and on days 1 and 2 post-operation.

### Statistical Analysis and Sample Size

#### Power analysis

We conducted a pilot study with eight patients who were undergone ESD for early gastric cancer after approval by the institutional ethics committee. Among eight patients, four patients were undergone ESD with irrigation, and another four were not. We calculated SD, E (effective size) and sample size as following.

SD (standard deviation): 4.724 E (effective size): From the search results of our pilot study, the average log bacterial count of the post-gastric irrigation gastric juice was 5.06 in the regular group and 1.77 in the clean group. There was a significant differences between two groups. Though there wasn’t any precedent study referring to irrigation with saline solution, the target number of 24 subjects per group was calculated based on G* Power (http://www.psycho.uni-duesseldorf.de/abteilungen/aap/gpower3/download-and-register) using the the α level 0.05, β level 0.2. Using the effective size of 0.8, the target number resulted in 25, and we referred the number. All values are presented as the mean±SD. The patient baseline characteristics were analyzed using the unpaired *t*-test and the χ2 test. The bacterial counts were converted to logarithmic displays to conduct comparative evaluations. The spontaneous pain level VAS scores were analyzed using Fisher’s exact test at a 2-tailed significance level of 5%. Values of *P*<0.05 were considered statistically significant. Data and statistical analyses were performed using GraphPad Prism version 5 for Windows (GraphPad Software, San Diego, CA, USA).

## Results

There were no significant differences in age, gender, resection location, operation time, resected specimen diameter, number of endoscope withdrawals, total normal saline volume used to wash the stomach during ESD (except for irrigation), WBC, CRP and BT before ESD between the two groups (Table 1).

**Table 1:** Basal parameters of enrolled patients.

	Regular Group (n = 25)	Clean Group (n = 25)	*P* values
Age(yrs)(mean±SD)	73.5±7.0	72.7±9.5	0.2*
Gender(M/F)	17/8	15/10	0.6**
Location(U/M/L)	7/10/8	6/10/9	0.5**
Operation time(min)	120.5±62.7	127.8±43.7	0.3*
Resected specimen(mm)	46.8±13.0	43.7±15.2	0.5*
Withdrawn No. of endoscope	4.8±1.3	5.1±0.9	0.4*
Non-irrigation saline volume to wash the stomach(ml)	126.1±45.2	119.8±13.6	0.3*
WBC before ESD(cells/µl)	5171±1549	5253±1159	0.7*
CRP before ESD(mg/dl)	0.62±1.38	0.64±1.21	0.5*
Body temperature before ESD(°C)	36.2±2.56	36.4±3.65	0.3*

* Unparied *t*-test,

** χ^2^ test.

**Figure 2:**
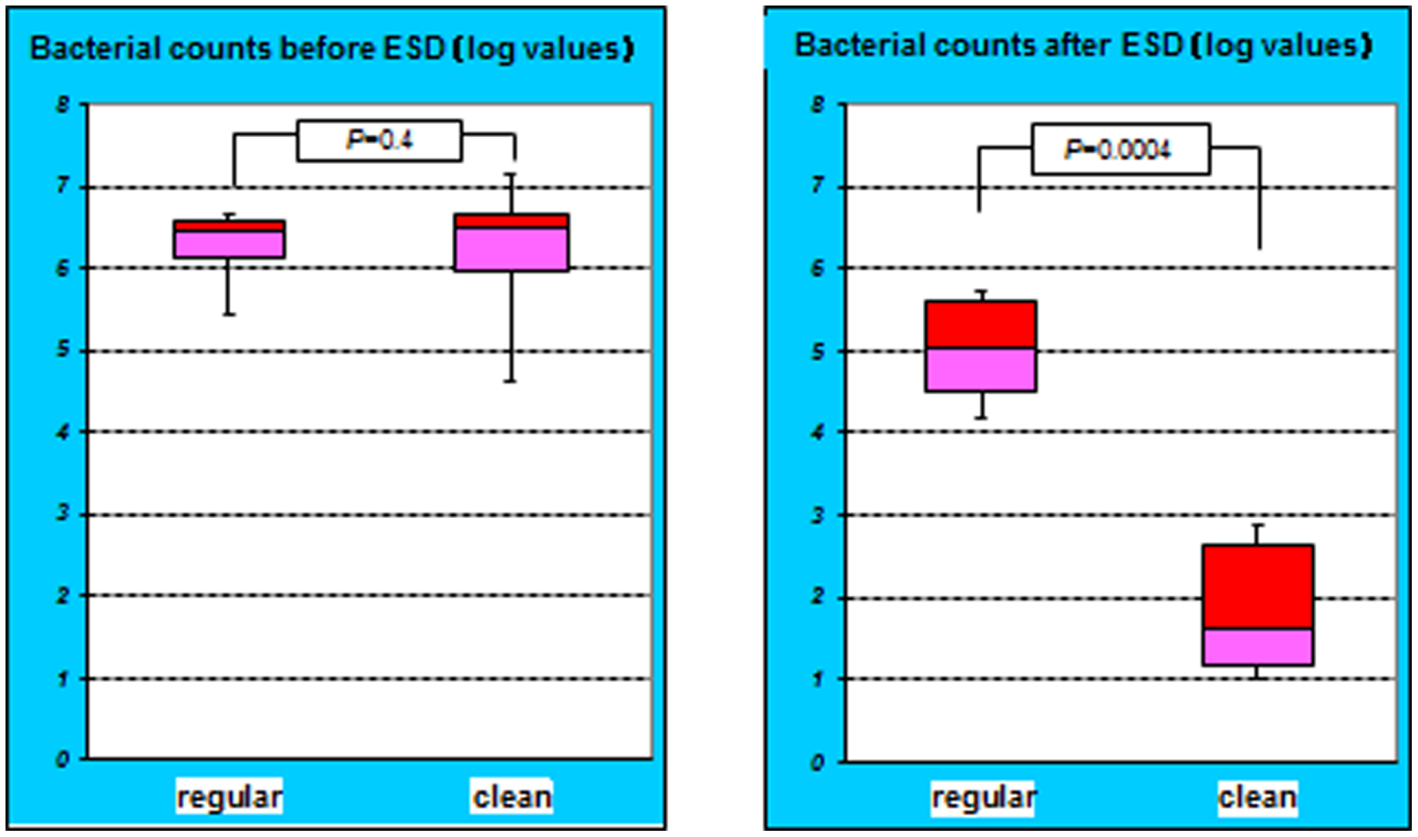
Comparison of logarithmic bacterial counts. The bacterial counts before ESD were not significantly different between the groups (*P = *0.4). However, the difference in bacterial counts after ESD was significant between the groups (*P = *0.0004).

In the regular group, the mean log bacterial count in pre-irrigation gastric juice was 6.26±0.58, while in the clean group; the mean log bacterial count of the pre-irrigation gastric juice was 6.20±1.09. The bacterial counts before ESD did not significantly differ between the groups (*P* = 0.4). However, the mean log bacterial count of the post-gastric irrigation gastric juice was 5.08±0.75 in the regular group and 1.86±0.86 in the clean group. The bacterial counts after ESD with gastric irrigation was significantly reduced in clean group compared to regular group (*P* = 0.0004) (Fig. 2).

**Figure 3:**
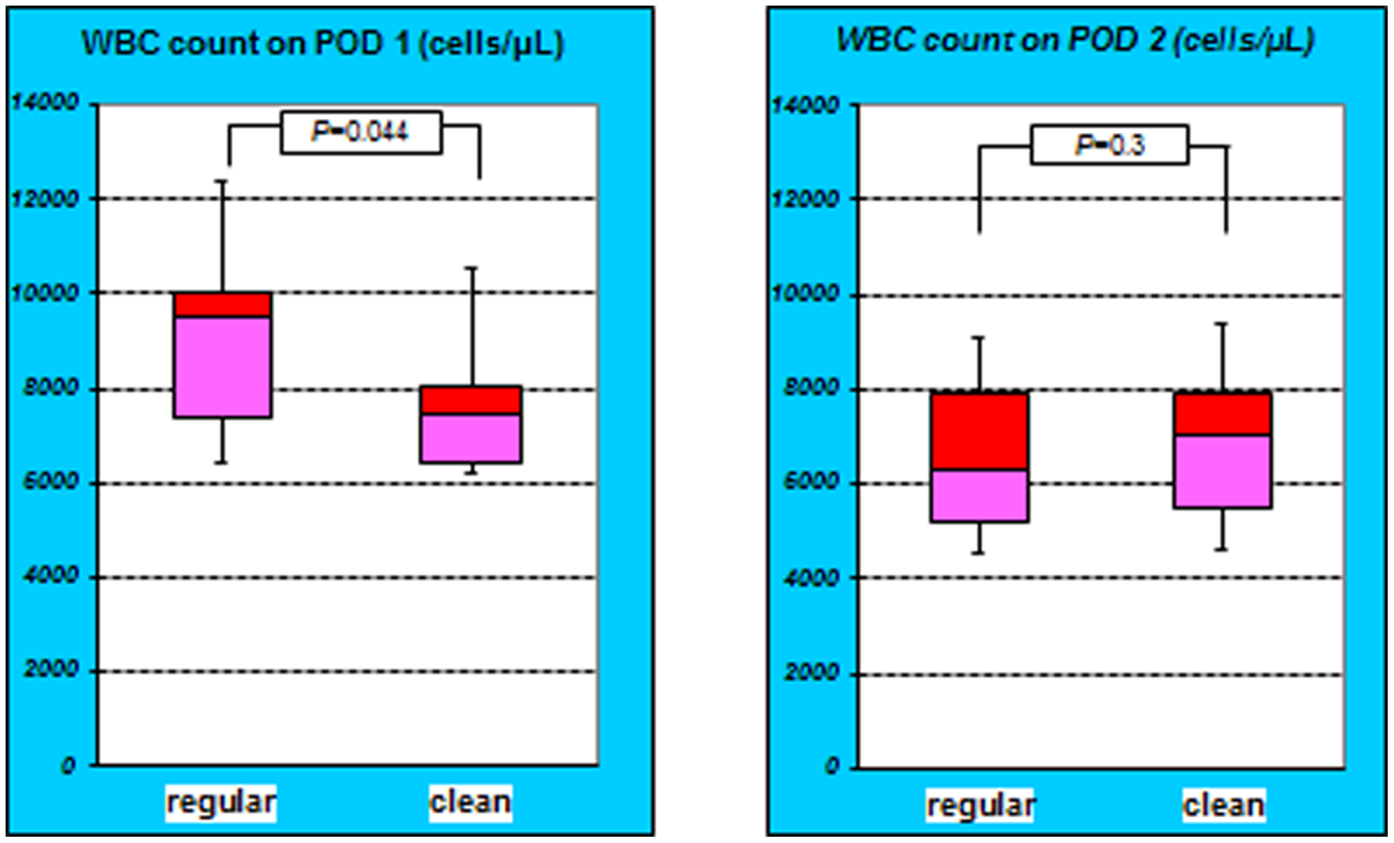
WBC counts between the group on PODs 1 and 2. The difference in the WBC counts on POD 1 was significant between the groups (*P = *0.044). However, on POD 2, the difference in the WBC counts on POD 2 was not significant (*P = *0.3). The WBC counts in the regular group on POD 1 were higher compared to clean group.

The mean WBC count in the regular group on POD 1 was 9903±4468. The mean WBC count in the clean group on POD 1 was 7735±2113. The difference in the mean WBC count on POD 1 was significant (*P* = 0.044). On the other hand, on POD 2 the mean WBC count in the regular group was 6658±1841, while it was 7055±2222 in the clean group. The difference in the mean WBC count on POD 2 was not significant (*P* = 0.3) (Fig. 3).

**Figure 4:**
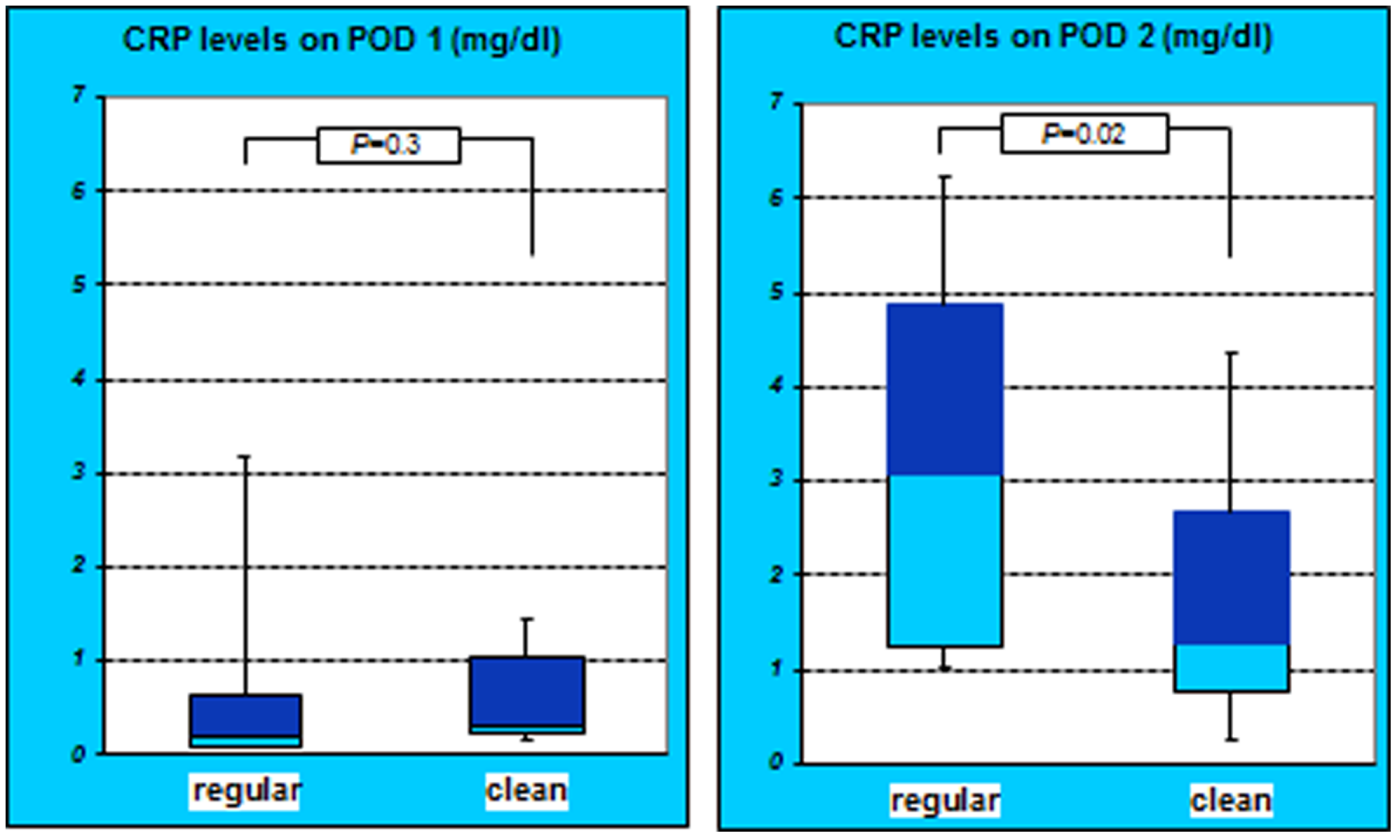
CRP level of both groups on POD 1 and 2. The difference in the CRP levels on POD 1 was not significant between the *2* groups (*P = *0.3). However, on POD 2, the difference in the CRP levels was significant (*P = *0.02). The CRP level in the regular group on POD 2 was higher compared to clean group.

The mean CRP level in the regular group on POD 1 was 0.94±1.71, while it was 0.8±1.19 in the clean group. The difference in the mean CRP level on POD 1 was not significant (*P* = 0.3). On POD 2, the mean CRP levels in the regular and clean groups were 3.44±2.47 and 1.80±1.64, respectively, and the difference was significant (*P* = 0.02) (Fig. 4).

**Figure 5:**
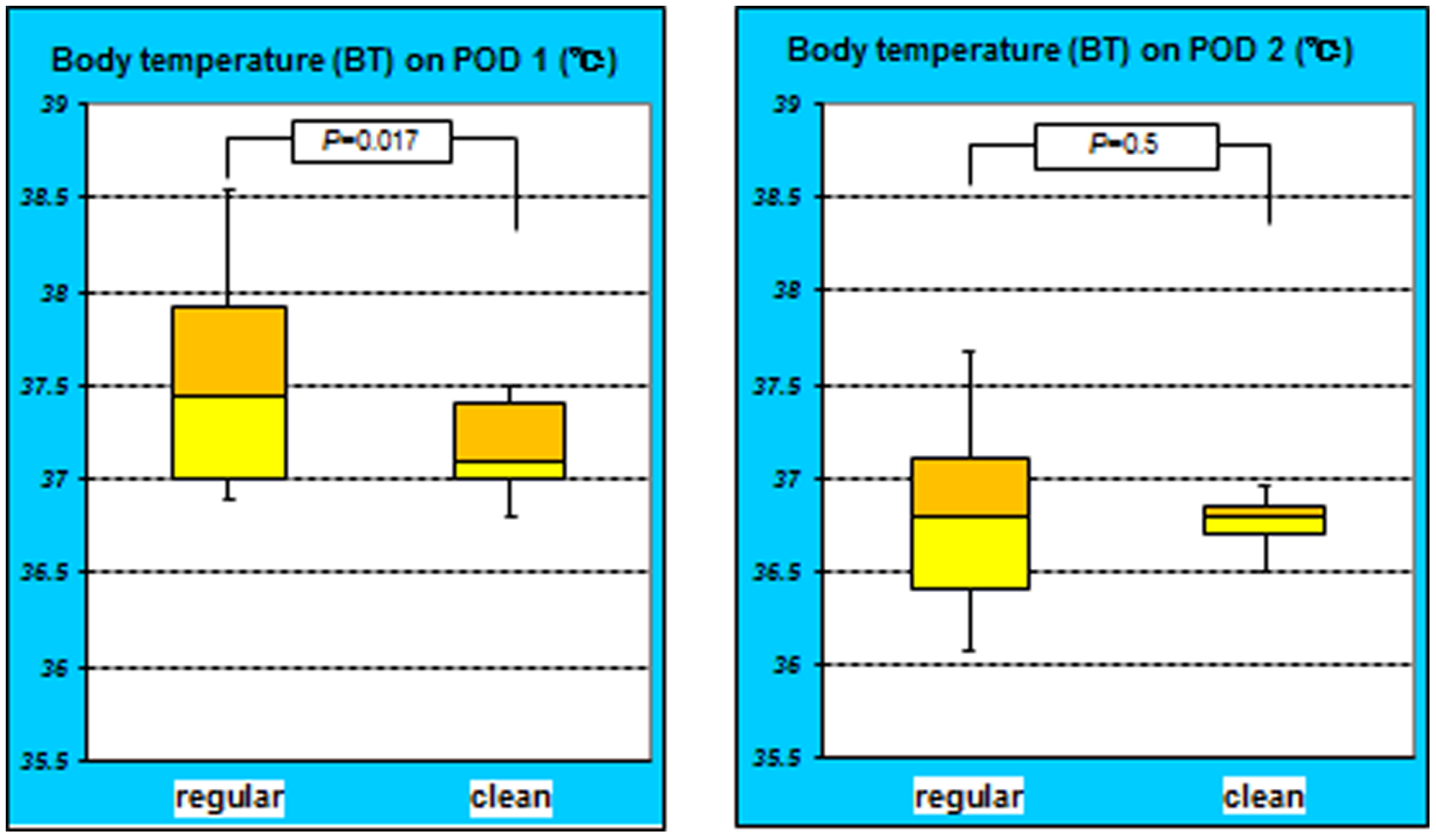
Time-dependent changes in body temperature of both groups. The difference in patient’s BT on POD 1 was significant between the groups (*P = *0.017). However, on POD 2, the BT did not significantly differ between the groups (*P = *0.5). The BT in the regular group on POD 1 was higher compared to clean group.

The mean BT in the regular group on POD 1 was 37.6±0.65, while it was 37.1±0.32 in the clean group. The difference in the mean BT on POD 1 was statistically significant (*P = *0.017). On POD 2, the mean BTs in the regular and clean groups were 36.8±0.76 and 36.7±0.22, respectively, which were not significantly different (*P = *0.5) (Fig. 5).

**Figure 6:**
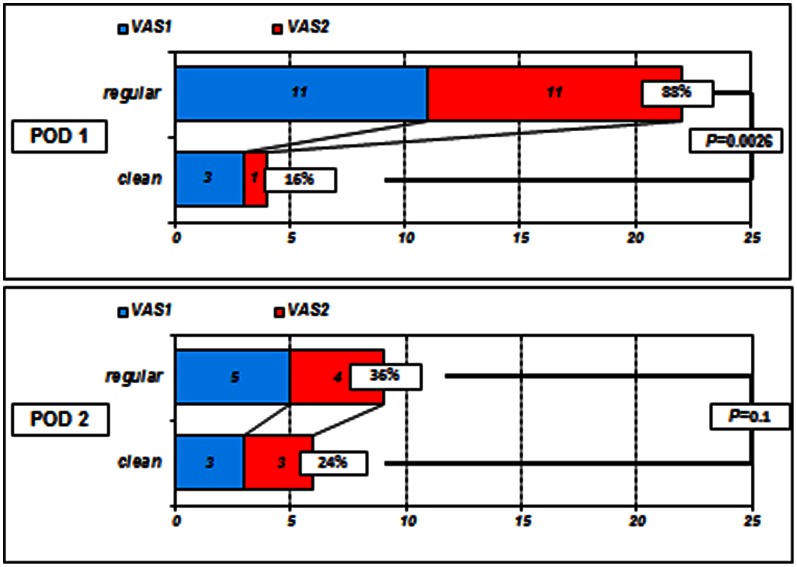
The proportion of VAS scores 1 to 2 between the groups on PODs 1 and 2. On POD 1, the proportion of patients in the regular group with mild to moderate spontaneous pain (VAS score 1 to 2) was higher than that of the clean group (*P = *0.0026). However, on POD 2, the proportions of patients with VAS scores 1 to 2 in both groups were similar and were not significant (*P = *0.1).

As shown in Figure 6, on POD 1 in the regular group, the proportion of patients with mild to moderate spontaneous pain (VAS score 1 to 2) was 88% versus 16% in the clean group (*P = *0.0026). However, on POD 2, the proportion of patients with mild to moderate spontaneous pain (VAS score 1 to 2) was 36% and 24% in the regular and clean groups, respectively (*P = *0.1) (Fig. 6).

## Discussion

Our results indicate that gastric irrigation significantly decreases the gastric bacterial count after ESD techniques. In the regular group, clinical evaluations on POD 1 revealed mild to moderate abdominal pain, and our analyses indicated that the increases in the WBC counts as well as BT were suppressed on POD 2. It is accepted that indigenous oral bacteria enter the stomach as a result of endoscope insertion, which may result in an increase in gastric bacteria and may cause a considerable risk of infection in the post-ESD artificial ulcer base.

When inserting a percutaneous endoscopic gastrostomy (PEG) tube, unchecked indigenous oral flora in the stomach may lead to fistula formation, infection and peritonitis, particularly after using the pull method. However, there are only a few reports of fistula, infection, or peritonitis complications using the push method [4].

While indigenous oral bacteria may become pathogenic infectious agents during ESD, it appears that gastric bacteria alone are not intra-abdominal infection causing pathogens after ESD, even in cases where gastric irrigation is not performed. The activity of indigenous oral bacteria may also increase as a result of an increased gastric pH after PPI administration. Patients who undergo ESD commonly take PPIs. PPI administration has been recommended to reduce post-ESD hemorrhage [5]. Normal gastric juice is generally known to have a pH of 3 or less, and almost no bacteria are able to propagate in the normal stomach. However, in elderly patients, it has been reported that the administration of H_2_-blockers and PPIs results in a sufficient increase in the pH of gastric juice as gastric acid secretion declines due to advancing age, which allows indigenous oral bacteria to infect the stomach and propagate there. This is sometimes observed in patients with feeding tubes. The decreased gastric pH reduces gastric bacterial counts [6], and irrigation with saline solution during endoscopic dissection procedures may potentially reduce or negate contamination of the stomach with indigenous oral bacteria. This may mitigate the effects of bacterial propagation resulting from PPI administration to prevent post-ESD hemorrhage.

In animal models of natural orifice transluminal endoscopic surgery (NOTES), it has been reported that indigenous oral bacteria may become pathogenic during NOTES and potentially cause intra-abdominal infection. One animal experiment indicated that inadequate disinfection was responsible for 9.6% of infectious complications [7]. A variety of methods for preventing infection following transgastric and transrectal NOTES have been tested in animal models involving preoperative disinfection from the oral cavity to the stomach, along with various methods for closing the affected areas. However, there are relatively few reports related solely to infection, and upto date, there have been no reports evaluating gastric irrigation before ESD in humans. When a perforation occurs during ESD, *Escherichia coli, Klebsiella pneumoniae, Streptococcus viridans, Pseudomonas aeruginosa, Staphylococcus aureus, Neisseria shallow, Streptococcus haemolyticus* and oral streptococci, can escape and cause pan-peritonitis.

In a recent animal study, gastric irrigation with a 10% povidone-iodine solution was performed following tubal ligation and cholecystectomy. Examinations performed 20 days following these procedures revealed no clear findings of intra-abdominal infection or abscess formation [8].

Irrigation with 500 mL of saline solution and 200 mL of 5% povidone-iodine (Betadine) solution diluted with purified water has been reported to reduce bacterial counts in gastric juice cultures from 15 to 17×10^3 ^CFU/mL to 0 to 3 CFU/mL, thereby suppressing post-NOTES adhesion and abscess formation [9]. In experiments using pigs, irrigation with 500 mL of saline solution has also been reported to significantly reduce bacterial counts in gastric juice culture during mesh placement for ventral hernia repair using a transgastric NOTES technique [10]–[12]. Although the validity of gastric irrigation with a povidone-iodine solution through a transgastric route in humans has been reported [13], [14], however, there has been no research on the systemic signs of infection or any other type of infection.

The effects of gastric disinfection in humans with 5% or higher concentrations of iodine solution on perioperative gastric mucosa injury and the gastric mucosa post-operation are currently unknown. The risks and benefits of antisepsis must always be considered, and gastric irrigation with 2 L of saline solution can be regarded as a safe and valid option for gastric disinfection after the introduction of oral bacteria via endoscope insertion during ESD procedures.

Our study results indicate that pre-ESD gastric irrigation reduces bacterial counts and removes necrotic agents and foreign bodies effectively. Pre-ESD gastric irrigation may also allow an intra-abdominal infection to be more easily suppressed without antibiotics, even if complications such as perforation occur.

In conclusion, the prevention of infection during the ESD procedure or perforation complications is an important issue for its clinical application. This study finding using 50 patients gave an indication that it is feasible and effective to use pre-ESD gastric irrigation to suppress infection in humans and led to favorable clinical outcomes.

## Supporting Information

Protocol S1 **Trial Protocol.**
(DOC)

Checklist S1 **CONSORT Checklist.**
(DOC)

Diagram S1 **CONSORT Flow Diagram.**
(DOC)
