# Supplementary material for: Effects of Gastric Irrigation on Bacterial Counts before Endoscopic Submucosal Dissection: A Randomized Case Control Prospective Study
Source: PLoS One. 2013 Jun 7;8(6):e65377. doi: 10.1371/journal.pone.0065377 (PMC3676410; doi:10.1371/journal.pone.0065377)
Supplement: Protocol S1 — Trial Protocol. (DOC) [file pone.0065377.s001.doc]

**Study Protocol**

***The Institutional Ethics Committee of Kagawa University Hospital***

**1750-1, Miki, Kida, Kagawa, Japan**

**Title: Effects of Gastric Irrigation on Bacterial Counts before Endoscopic Submucosal Dissection**

**Researchers**

Hirohito Mori, MD, PhD

Hideki Kobara, MD, PhD

Noriko Nishiyama, MD

Shintaro Fujihara, MD

Makoto Oryu, MD, PhD

Tsutomu Masaki, MD, PhD

Departments of Gastroenterology and Neurology, Faculty of Medicine, Kagawa University, Kagawa, Japan

**Background and aims**

The search for the most advanced means of surgical disinfection is on-going, and methods reported in recent years include scrubbing with sterile water using a sterile culture brush to disinfect the skin and waterless rubbing using regular soap and fast-drying disinfectant, and these methods are recommended by the United States Centers for Disease Control (CDC). During surgical manoeuvres such as skin incision in the approach to target organs, indigenous bacteria, mycobacterium tuberculosis, filamentous fungi, spore-forming bacteria, and viruses are all potentially pathogenic and can cause infections. During endoscopic submucosal dissection (ESD), an endoscope is inserted into the stomach through the mouth, which results in inevitable exposure of the dissection site to oral bacteria. However, no reports exist concerning gastric irrigation and oral antisepsis in ESD. Because intraluminal treatment in the stomach is a semi-closed system where gastric acid is present, there the validity of oral antisepsis with isodine or irrigation with saline solution in ESD has yet to be confirmed, although there are many reports concerning the use of isodine and saline irrigation in natural orifice transluminal endoscopic surgery (NOTES).

A frequent complication of ESD is perforation of the gastric wall, which can often be treated with conservative therapy by closing the perforation with a clip. If it is proven that gastric irrigation can reduce bacteria counts without injuring gastric mucosa, it can then be expected that intra-abdominal infection might be more easily suppressed with antibiotics, even if complications such as perforation occur. Gastric wall perforation may also result in postoperative complication in NOTES procedures, and animal experiments with transgastric NOTES procedures have been used as a method of predicting intra-abdominal infections after endoscopic perforations. The present study was a prospective randomized controlled trial of the effects of gastric irrigation with 2 L saline solution during ESD on gastric bacteria counts, and we have also included a review of a series of hybrid NOTES cases in which validity of such irrigation was examined.

**Methods**

A pilot study with 8 patients who underwent ESD for early gastric cancer after receiving approval from the institutional ethics committee. Among the 8 patients, 4 patients were irrigated with 2 L of saline solution and 4 were not.

Calculated the sample size from the pilot study.

An opaque envelope method was used to randomly divide the subjects into a clean group, in which irrigation was performed, and a regular group (irrigation was not performed). Each group contained 25 patients.

All of the patients began a 30 mg daily dose of a proton-pump inhibitor (esomeprazole) on the day prior to ESD.

The randomization was achieved using sealed, numbered envelopes, as prepared previously by Dr. M. O.

The randomization code was not broken until the study was completed.

ESD was performed by 1 of 5 endoscopists. None of the endoscopists were informed of this study.

Each endoscopist was informed on how to perform the irrigation and was blinded to the randomization process to avoid any bias.

All bacterial cultures were performed by a bacteriologist (Dr. N. N.) who was blinded to the randomization process and did not know which patients received irrigation.

At the end of the study, the data were analyzed in a blinded manner.

**Procedures**

At the beginning of the ESD procedure, 20 mL of distilled water was dispersed onto the gastric wall, and 20 mL of gastric juice was collected in a sterile culture tube for evaluation as the pre-procedure bacterial culture (37°C, 48 hours). Irrigation was performed throughout the stomach with 2 L of saline solution using a water jet attached to an endoscope. After completion of the ESD and recovery of the resected tumor, 20 mL of distilled water was again dispersed onto the gastric wall, and 20 mL of gastric juice was collected in a sterile culture tube as the post-ESD culture. The sterile culture tube was passed through the working channel. We counted the number of times the endoscope was withdrawn and recorded the normal saline volume that was used to wash the stomach during ESD (except for irrigation). White blood cell (WBC) counts and C-reactive protein (CRP) levels were measured the day before ESD and on days 1 and 2 post-ESD. The body temperature (BT) was checked the day before ESD and on days 1 and 2 post-ESD.

**An assessment of spontaneous abdominal pain**

4-level visual analog scale (VAS): VAS-0 represented no spontaneous pain, 1 represented mild spontaneous pain, 2 represented moderate spontaneous pain, and 3 represented severe spontaneous pain.

**Study design;** randomized prospective study

**Primary outcome**

The primary outcome was the difference in pre-ESD gastric juice culture bacterial counts in the clean group versus the regular group.

**Secondary outcomes**

The secondary outcomes included the following:

1. WBC, CRP and BT values on days 1 and 2 post-operation
2. Spontaneous pain level VAS scores just after the operation and on days 1 and 2 post-operation.

**Setting;** Single center

**Patients**

- This prospective clinical study was conducted with pre-approval by *the institutional ethics committee of Kagawa university hospital, Kagawa,* Japan and was *enrolled with the university hospital medical information network (UMIN) #000008691.* And the study began after we obtained informed consent to patients by written form.
- **Exclusion criterion;**　complication during ESD

**Study period**；From June 2012 to March 2013

**References**

1.Use of flexible endoscopes for NOTES: sterilization or high-level disinfection? [Surg Endosc.](http://www.ncbi.nlm.nih.gov/pubmed?term=Surgical endoscopy 2010%3B24%3A1581-1588) 2010 Jul;24(7):1581-8. Epub 2009 Dec 24.

[Spaun GO](http://www.ncbi.nlm.nih.gov/pubmed?term=Spaun GO%5BAuthor%5D&cauthor=true&cauthor_uid=20033708), [Goers TA](http://www.ncbi.nlm.nih.gov/pubmed?term=Goers TA%5BAuthor%5D&cauthor=true&cauthor_uid=20033708), [Pierce RA](http://www.ncbi.nlm.nih.gov/pubmed?term=Pierce RA%5BAuthor%5D&cauthor=true&cauthor_uid=20033708), [Cassera MA](http://www.ncbi.nlm.nih.gov/pubmed?term=Cassera MA%5BAuthor%5D&cauthor=true&cauthor_uid=20033708), [Scovil S](http://www.ncbi.nlm.nih.gov/pubmed?term=Scovil S%5BAuthor%5D&cauthor=true&cauthor_uid=20033708), [Swanstrom LL](http://www.ncbi.nlm.nih.gov/pubmed?term=Swanstrom LL%5BAuthor%5D&cauthor=true&cauthor_uid=20033708)

2. Can gastric irrigation prevent infection during NOTES mesh placement

[J Gastrointest Surg.](http://www.ncbi.nlm.nih.gov/pubmed/18704595) 2008 Nov;12(11):2010-4. Epub 2008 Aug 13.

[Buck L](http://www.ncbi.nlm.nih.gov/pubmed?term=Buck L%5BAuthor%5D&cauthor=true&cauthor_uid=18704595), [Michalek J](http://www.ncbi.nlm.nih.gov/pubmed?term=Michalek J%5BAuthor%5D&cauthor=true&cauthor_uid=18704595), [Van Sickle K](http://www.ncbi.nlm.nih.gov/pubmed?term=Van Sickle K%5BAuthor%5D&cauthor=true&cauthor_uid=18704595), [Schwesinger W](http://www.ncbi.nlm.nih.gov/pubmed?term=Schwesinger W%5BAuthor%5D&cauthor=true&cauthor_uid=18704595), [Bingener J](http://www.ncbi.nlm.nih.gov/pubmed?term=Bingener J%5BAuthor%5D&cauthor=true&cauthor_uid=18704595).
