# Supplementary material for: Effects of Gastric Irrigation on Bacterial Counts before Endoscopic Submucosal Dissection: A Randomized Case Control Prospective Study
Source: PLoS One. 2013 Jun 7;8(6):e65377. doi: 10.1371/journal.pone.0065377 (PMC3676410; doi:10.1371/journal.pone.0065377)
Supplement: Checklist S1 — CONSORT Checklist. (DOC) [file pone.0065377.s002.doc]

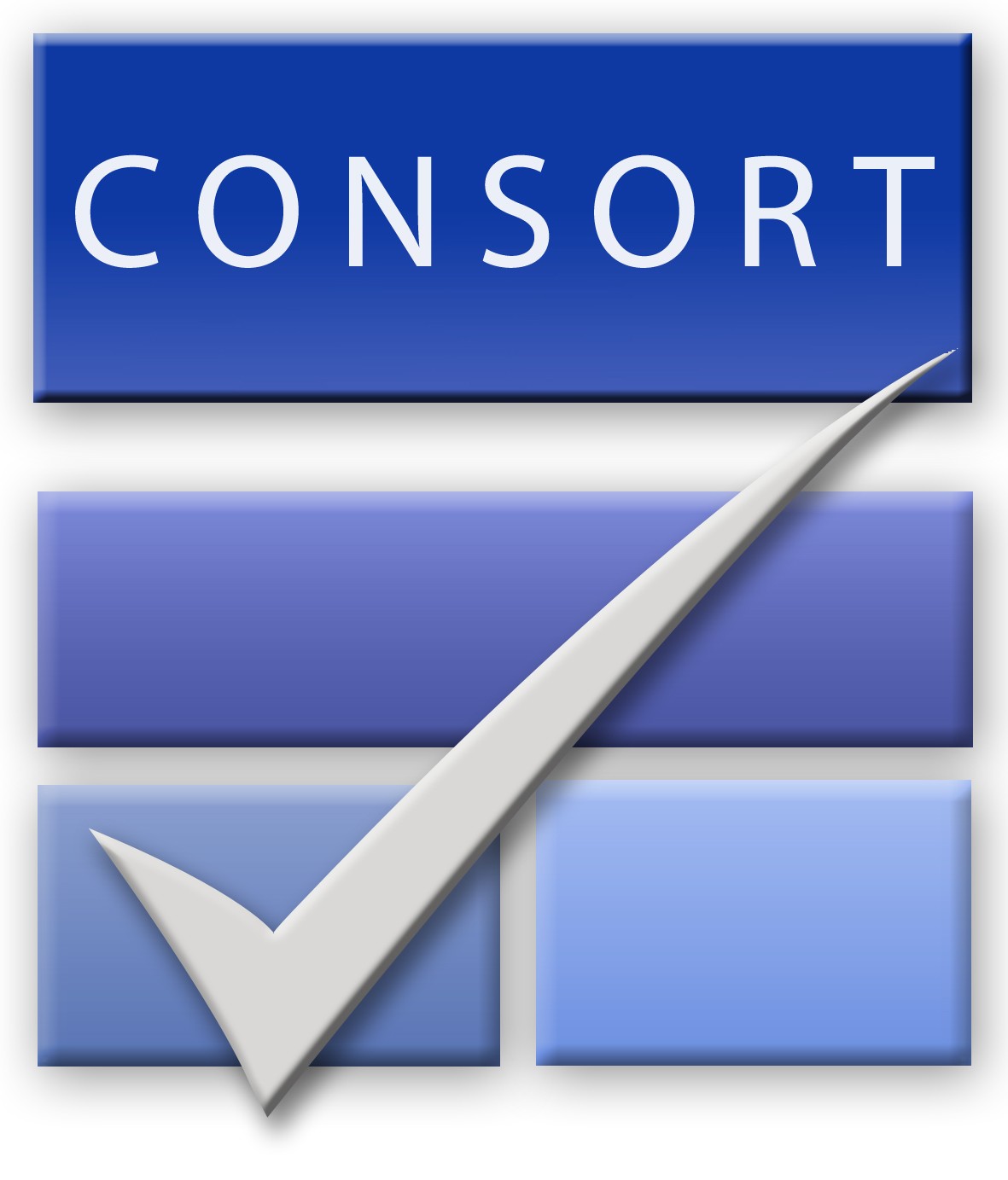
Checklist S1

| Section/Topic | Item No | Checklist item | Reported Section |
| --- | --- | --- | --- |
| Title and abstract | | | |
|  | 1a | Identification as a randomised trial in the title | Title section |
|  | 1b | Structured summary of trial design, methods, results, and conclusions (for specific guidance see CONSORT for abstracts) | Abstract section |
| Introduction | | | |
| Background and objectives | 2a | Scientific background and explanation of rationale | Introduction section |
|  | 2b | Specific objectives or hypotheses | Patients and Methods section |
| Methods | | | |
| Trial design | 3a | Description of trial design (such as parallel, factorial) including allocation ratio | Patients and Methods section |
|  | 3b | Important changes to methods after trial commencement (such as eligibility criteria), with reasons | Patients and Methods section |
| Participants | 4a | Eligibility criteria for participants | Patients and Methods section |
|  | 4b | Settings and locations where the data were collected | Patients and Methods section |
| Interventions | 5 | The interventions for each group with sufficient details to allow replication, including how and when they were actually administered | Patients and Methods section |
| Outcomes | 6a | Completely defined pre-specified primary and secondary outcome measures, including how and when they were assessed | Outcomes in Patients and Methods section |
|  | 6b | Any changes to trial outcomes after the trial commenced, with reasons | - |
| Sample size | 7a | How sample size was determined | Statistical analysis and Sample size  In Patients and Methods section |
|  | 7b | When applicable, explanation of any interim analyses and stopping guidelines | - |
| Randomisation: |  |  |  |
| Sequence generation | 8a | Method used to generate the random allocation sequence | Patients and Methods section |
|  | 8b | Type of randomisation; details of any restriction (such as blocking and block size) | Patients and Methods section |
| Allocation concealment mechanism | 9 | Mechanism used to implement the random allocation sequence (such as sequentially numbered containers), describing any steps taken to conceal the sequence until interventions were assigned | Patients and Methods section |
| Implementation | 10 | Who generated the random allocation sequence, who enrolled participants, and who assigned participants to interventions | Patients and Methods section |
| Blinding | 11a | If done, who was blinded after assignment to interventions (for example, participants, care providers, those assessing outcomes) and how | Patients and Methods section |
|  | 11b | If relevant, description of the similarity of interventions | - |
| Statistical methods | 12a | Statistical methods used to compare groups for primary and secondary outcomes | Ethical statement in Patients and Methods section |
|  | 12b | Methods for additional analyses, such as subgroup analyses and adjusted analyses | Statistical analysis and Sample size  In Patients and Methods section |
| Results | | | |
| Participant flow (a diagram is strongly recommended) | 13a | For each group, the numbers of participants who were randomly assigned, received intended treatment, and were analysed for the primary outcome | Statistical analysis and Sample size  In Patients and Methods section |
|  | 13b | For each group, losses and exclusions after randomisation, together with reasons | Statistical analysis and Sample size  In Patients and Methods section |
| Recruitment | 14a | Dates defining the periods of recruitment and follow-up |  |
|  | 14b | Why the trial ended or was stopped |  |
| Baseline data | 15 | A table showing baseline demographic and clinical characteristics for each group | Table 1 |
| Numbers analysed | 16 | For each group, number of participants (denominator) included in each analysis and whether the analysis was by original assigned groups | Results section or Table 1 |
| Outcomes and estimation | 17a | For each primary and secondary outcome, results for each group, and the estimated effect size and its precision (such as 95% confidence interval) | Outcomes in Patients and Methods section |
|  | 17b | For binary outcomes, presentation of both absolute and relative effect sizes is recommended | Patients and Methods section |
| Ancillary analyses | 18 | Results of any other analyses performed, including subgroup analyses and adjusted analyses, distinguishing pre-specified from exploratory | Statistical analysis and Sample size in Methods section |
| Harms | 19 | All important harms or unintended effects in each group (for specific guidance see CONSORT for harms) | Results section |
| Discussion | | | |
| Limitations | 20 | Trial limitations, addressing sources of potential bias, imprecision, and, if relevant, multiplicity of analyses | Discussion section |
| Generalisability | 21 | Generalisability (external validity, applicability) of the trial findings | Discussion section |
| Interpretation | 22 | Interpretation consistent with results, balancing benefits and harms, and considering other relevant evidence | Discussion section |
| Other information | | |  |
| Registration | 23 | Registration number and name of trial registry | Abstract section |
| Protocol | 24 | Where the full trial protocol can be accessed, if available | Patients and Methods section |
| Funding | 25 | Sources of funding and other support (such as supply of drugs), role of funders | Acknowledgments in discussion section |

*We strongly recommend reading this statement in conjunction with the CONSORT 2010 Explanation and Elaboration for important clarifications on all the items. If relevant, we also recommend reading CONSORT extensions for cluster randomised trials, non-inferiority and equivalence trials, non-pharmacological treatments, herbal interventions, and pragmatic trials. Additional extensions are forthcoming: for those and for up to date references relevant to this checklist, see [www.consort-statement.org](http://www.consort-statement.org/).
