# Supplementary material for: Effects of Gastric Irrigation on Bacterial Counts before Endoscopic Submucosal Dissection: A Randomized Case Control Prospective Study
Source: PLoS One. 2013 Jun 7;8(6):e65377. doi: 10.1371/journal.pone.0065377 (PMC3676410; doi:10.1371/journal.pone.0065377)
Supplement: Diagram S1 — CONSORT Flow Diagram. (DOC) [file pone.0065377.s003.doc]

**CONSORT Flow Diagram**

**Allocation**

**Analysis**

**Follow-Up**

**Enrollment**

Assessed for eligibility (n=50)

Excluded (n=0)

♦  Not meeting inclusion criteria (n=0)

♦  Declined to participate (n=0)

♦  Other reasons (n= 0)

Analysed (n=25)
♦ Excluded from analysis (give reasons) (n=0)

Lost to follow-up (give reasons) (n=0)

Discontinued intervention (give reasons) (n=0)

Allocated to intervention (n=25)

♦ Received allocated intervention (n=25)

♦ Did not receive allocated intervention (give reasons) (n=0)

Lost to follow-up (give reasons) (n=0)

Discontinued intervention (give reasons) (n=0)

Allocated to intervention (n=25)

♦ Received allocated intervention (n=25)

♦ Did not receive allocated intervention (give reasons) (n=0)

Analysed (n=25)
♦ Excluded from analysis (give reasons) (n=0)

Randomized (n=50)
